# A new TK model approach to assess the effect of migration on copper toxicokinetics in inbred populations of the flour beetle, *Tribolium castaneum*

**DOI:** 10.1007/s00128-017-2093-7

**Published:** 2017-05-26

**Authors:** Sebastian Żmudzki, Natnael T. Hamda, Patrycja Gibas - Tybur

**Affiliations:** 1 Institute of Environmental Sciences, Jagiellonian University, Gronostajowa 7, 30-387, Kraków, Poland; 2 Department of Fisheries, Wildlife, and Conservation Biology, University of Minnesota, St. Paul, MN 55108 USA

**Keywords:** Copper tolerance, TK model, Copper adaptation, Midgut physiology

## Abstract

The aim of the study was to determine the influence of migration on copper (Cu) kinetics of male *Tribolium castaneum* after 25 generations of exposure for Cu-adapted and non-Cu-adapted inbred lines. Adapted lines were kept on a flour medium contaminated with 1000 mg Cu kg^−1^. A medium contaminated with 2000 mg Cu kg^−1^ of copper was used during the intoxication phase. Based on our data and literature reports, we introduced a new two-phase four-stage toxicokinetics (TK) model. The intoxication phase was successfully divided into three stages with separate assimilation rate constant (k_a_) and elimination rate constant (k_e_) values. The influence of migration was examined by comparing k_a_ and k_e_ parameters confidence intervals. In non-contaminated environments, migrants significantly increased k_a_ and k_e_ values in the second stage. Migrants decreased the maximum Cu accumulation observed in the experiment. The results indicated that the TK model must show high goodness-of-fit to be a useful tool for comparing treatments.

**Electronic supplementary material:**

The online version of this article (doi:10.1007/s00128-017-2093-7) contains supplementary material, which is available to authorized users.

Anthropogenic activity has massively changed natural environments. Large amounts of natural elements, including trace metals, have been relocated from deep deposits to surface soil layers and water bodies (Newman 2009).

One of the most abundant metals in the environment is copper. Cu is an essential element that is responsible for many metabolic processes, but is toxic at high doses. It is introduced to the environment in large quantities through fossil fuels combustion, mining and smelting activities, and through the application of fertilizers, sewage sludge, algicides, fungicides and molluscicides (Flemming and Trevors 1989). Multi-generational exposure to elevated levels of metals can be a selection factor and can lead to the evolution of adaptations that enable organisms to cope with elevated metal levels in the environment (Posthuma et al. 1992).

A second important problem caused by human activity is habitat fragmentation. It can pose a serious threat to the survival of animal populations and lead to inbreeding (Reed 2004) and inbreeding depression manifested by decreases in productivity, the viability of individuals, lifespan, and finally in population size decrease. The co-occurrence of habitat fragmentation and metal pollution is common across urbanized and industrialized areas. Limited population sizes and rapid selection for increased resistance to pollutants lead to a decrease of genetic diversity and can intensify inbreeding depression (Armbruster 2005). The threat of local population extinction stemming from small population size and inbreeding can be mitigated by immigrations (Carlson et al. 2014). Immigrants can significantly elevate heterozygosity and the vitality of progeny however, immigrants arriving from different environments that do not bear beneficial adaptations can also negatively affect small populations adapted to pollution or local microhabitats (Lopez et al. 2009).

In polluted environments, one of the primary factors that determine an individual’s performance is its toxicokinetics (TK) response. It provides a general overview of the physiology of organisms with respect to toxicant management, detoxification, acclimation or adaptation and the efficiency of defense mechanisms (Łagisz et al. 2005; Posthuma and Van Straalen 1993; Postma et al. 1996), or differences between species (Janssen et al. 1991). The objective of this study was to assess the influence of migrations between small, inbred Cu-adapted and non-adapted lines of *Tribolium castaneum*on on Cu TK. The main questions were: do migrants cause heterosis, adaptation additivity or outbreeding depression and evolutionary rescue reduction in the first generation after migrations? Are these effects different in Cu-adapted and non-adapted populations?

## Materials and Methods

The research was based on 200 inbred lines of the flour beetle *T. castaneum* derived from a multi-generational selection experiment conducted for 25 generations in our laboratory. The lines were established from a highly polymorphic stock culture created by breeding individuals (for three generations) from cultures obtained from 12 laboratories around the world. From the stock culture, 25 individuals in the pupa stage were randomly sampled for each selection line and placed in vials filed with 5 g of flour medium for 18 days. Successfully emerged 20 adults were randomly selected to establish each line. They were placed in vials filed with 20 g of medium and laid eggs for three days. Eggs were incubated in 30°C, after 21 days emerged pupae sampled (25 individuals) and this procedure was repeated for the next generation and repeated for all subsequent generations. The experimental lines were divided into two groups; 100 lines were kept in control selection environment (i.e., raised on non contaminated medium) and 100 lines were raised in copper contaminated selection environment (1000 mg kg^−1^ of Cu in medium). Calculated increase in the inbreeding coefficient over time, after 25 generations of isolation was ΔF = 0.47 (Soule and Wilcox 1980). Preliminary studies (unpublished data) confirmed that there were significant inheritable differences between inbred populations selected and non-selected for Cu resistance.

After 22 generations, 20 lines were selected for the study from among the 200 inbred lines: ten of them from lines kept in non-contaminated selection environment and the other 10 from lines kept in Cu-contaminated selection environment. The criterion of the selection was resistance to copper contamination measured by reproduction rate (the number of emerged adult progeny) in non-contaminated environment and environment contaminated with 3000 mg kg^−1^ Cu).

**Fig. 1:**
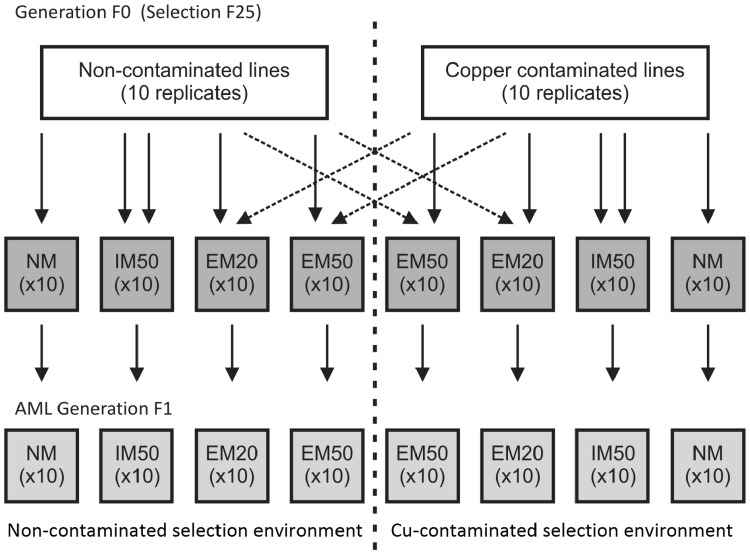
Design of the migration experiment. After-migration lines (*AML*): *NM* no migrations, *IM50* internal migrations (50% migrants from the same selection environment), *EM20* external migrations (20% migrants from different selection environment), *EM50* external migrations (50% migrants from different selection environment)

After 25 generations of selection on each of the 20 lines chosen for the experiment, the founders of the next generation were randomly sampled in the pupa stage: 50 males and 50 females were incubated separately. After 21 days of incubation, mature animals from each line were assigned to one of seven different migration treatments: ‘after-migration lines’ (AML) with no migrants (NM—control lines maintained under the same regime as in the main selection experiment), lines with 50% ‘internal’ migrants (IM50—migrants taken from a randomly chosen line from the same selection environment), and lines with either 20% or 50% of ‘external’ migrants (EM20 and EM50—migrants derived from a line from different selection environment) (Fig. 1). According to the experiment setup, animals were assigned to the AML in adequate number in the same type of selection environment (residual individuals) or moved as migrants to the second selection environment (Fig. 1). Thus, 10 replicates per group (treatment) were established. Individuals assigned to a treatment were mixed (residual individuals and migrants) in 50 mL vials filed with appropriate medium. In each case there were 20 fertile founders with a sex ratio of 1:1. The beetles were kept on the same medium type as the ancestral population (50 g medium, 7 days of egg deposition). The F1 progeny were used for the TK experiment.

For kinetics experiment we randomly sampled 105 males in the pupa stage from each AML and incubated them. Mature animals, aged 41–49 days from oviposition were used in TK experiment. We allocated them into 100 mL plastic containers filled with medium contaminated according to kinetic experiment phase. The experiment was divided into two phases. During the contamination phase (28 days) the animals were kept on Cu-contaminated medium (2000 mg kg^−1^). Then they were switched onto the control medium at the start of the decontamination phase (12 days). The animals from the experimental cultures were sampled on the following days, determined from the start of the contamination phase: 0, 0.5, 1, 1.5, 2, 4, 6, 8, 12, 16, 20, 24 and 28. We used the same regime during the decontamination phase (sample days: 28.5, 29, 29.5, 30, 32, 34, 36 and 40). Each sample consisted of three individuals. The collected animals were starved for 24 h to empty the gut of contents, euthanized by freezing, and stored in a frozen state. For analyses of Cu concentration, the samples were prepared and analyzed according to standardized method (Bednarska and Stępień 2015). A reference material (Bovine liver SRM 1577c, NIST) was analyzed for eight replicates. The recovery for Cu (mean% ± SD) was 100.7% ± 4.48%.

All experimental cultures were maintained in conditions recommended for *T. castaneum* (Sokoloff 1974). The Cu-contaminated medium was obtained by diluting CuCl_2_ (Copper (II) Chloride Dihydrate Pure p.a., POCH) in deionized water (300 mL kg^−1^of flour). The flour mixture was dried at 105°C for 24 h and powdered using a laboratory mill (MUKF 10 type, Młynpol, Poland). The control medium was prepared using an identical procedure, but deionized water was used in place of the CuCl_2_ solution. To ascertain the concentrations of Cu in the medium the samples were prepared and analyzed according to standardized method (Bednarska and Stępień 2015). Five samples of the reference material (*Ulva lactuca* No 483 by CBR), analyzed together with the medium, gave a mean recovery (±SD) of 92.78% ± 1.02%. The Cu concentrations (mg kg^−1^) that we measured in the medium matched expected values (mean ± SD): control, 4.95 ± 0.11; nominal 2000 mg kg^−1^, 2055.19 ± 4.89.

The core assumptions of used model were that the uptake rate was proportional to the environmental concentration, and the elimination rate was proportional to the internal concentration, as described by Atkins (1969):

1 $$\frac{{d{C_i}}}{{dt}}={k_a}{C_e} - {k_e}{C_i}$$

where C_e_ is the external environmental Cu concentration (mg kg^−1^), C_i_ the internal body Cu concentration (mg kg^−1^), k_a_ is the assimilation rate constant (day^−1^), and k_e_ is the elimination rates constant (day^−1^). Solving Eq. 1, the internal Cu concentration at time t is expressed as follows:

2 $${{C_i}\left( t \right)={C_{i,{t_c}}} \cdot {e^{ - {k_e} \cdot \left( {t - {t_c}} \right)}}+{C_e}\frac{{{k_a}}}{{{k_e}}}\left( {1 - {e^{ - {k_e}\left( {t - {t_c}} \right)}}} \right)}$$

where C_i_ represents the internal Cu concentration at the start of each stage (mg kg^−1^), and t_*c*_ is the time at which one stage transitions to the next (day). In the first stage t_c_ = 0 and C_*i*_, *t*_*c*_ = C_0_ (i.e., the initial body Cu concentration at t_0_, mg kg^−1^). Outliers for each time point were detected with Q95% Dixon’s Test and excluded from further analyses. After removing the outliers, the geometric mean recommended for TK modeling (Laskowski et al. 2010) was calculated for each time point and all models were fit to these calculated means (C_i_, t_c_). The elimination and assimilation constants and their 95% confidence intervals (CI) were estimated using a program code developed in MATLAB (Version 8.0.0.783, R2012b). We compared the experimental groups (treatments) using the estimated confidence intervals. The chosen method is conservative and detects differences between treatments with high robustness.

## Results and Discussion

**Fig. 2:**
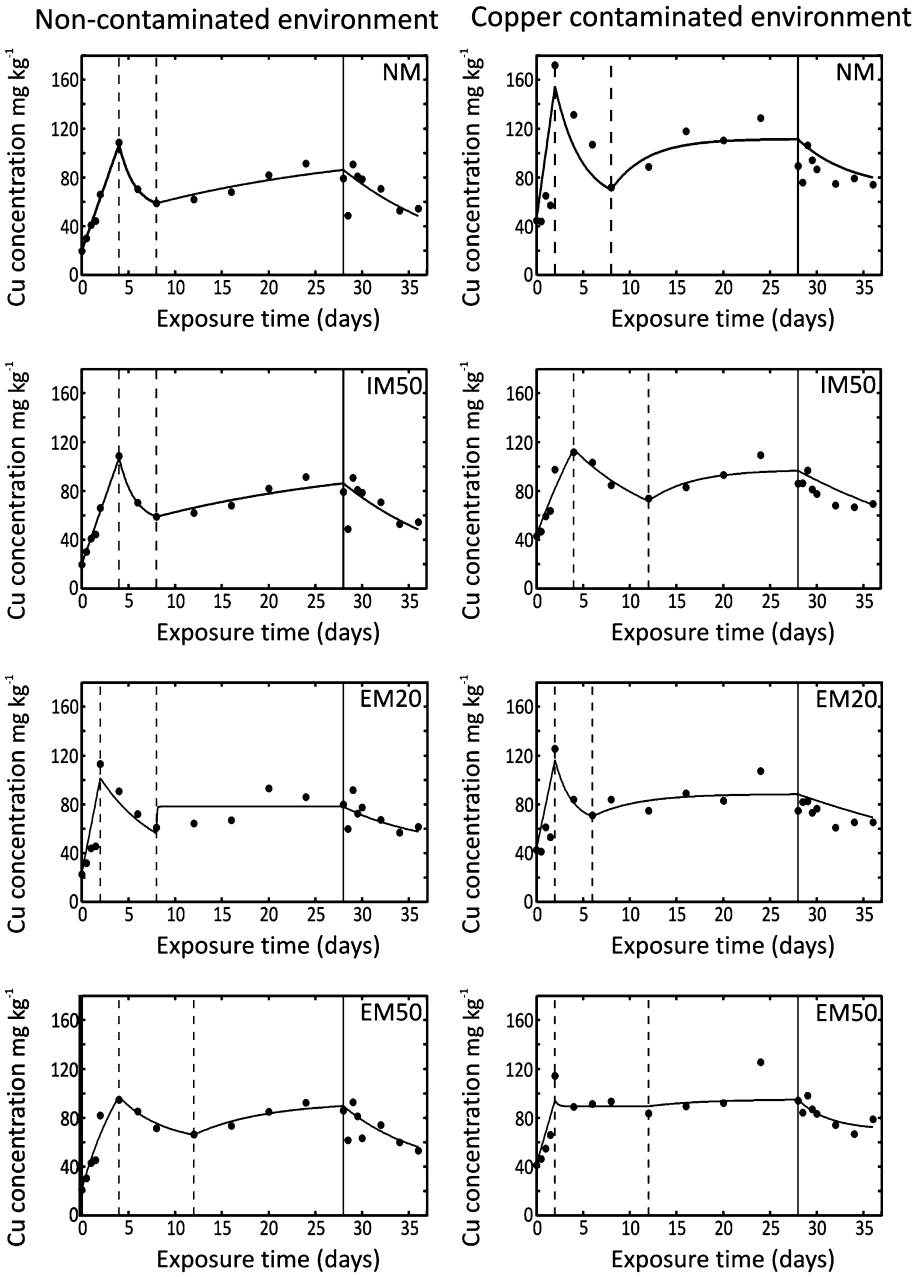
Copper kinetics in *T. castaneum* described by a two-phase, four-stage model in the experiment simulating migration between Cu-adapted and non-adapted populations

Insects responded with distinct metal uptake upon external metal exposure during the intoxication phase, and in all eight treatments (Fig. 2); they eliminated metals significantly during the elimination phase. The data for all time points, after removal of outliers, were normally distributed. The last time point (day 40) was not used for estimation of model parameters because the Cu concentrations on that day showed extreme deviation from the observed pattern, suggesting an analytical error.

Visual data analysis revealed that the classic two-phase model (Atkins 1969) does not adequately describe the observed pattern. Following the observed pattern in data (Fig. 2), we proposed a new TK approach. We divided the intoxication phase into three distinct stages reflecting changes in organism physiology, with k_a_ and k_e_ rates estimated individually for each stage and numbered accordingly from 1 to 3 with subjectively demarcated breakpoints. For the first stage, we used the initial Cu concentration measured in the beetles at t_0_ (C_0_). The initial concentrations for the subsequent stages were calculated from the model at the time of transition between two consecutive stages (Fig. 2; Tables 1, 2). Stage 1 (accumulation stage) was characterized by a rapid increase in Cu concentration from time 0 to the time during the experiment at which the organisms exhibited the highest body Cu concentrations. In stage 2 (depuration stage) the organisms exhibited a decrease in Cu level. The decrease extended from the maximum concentration reached at the end of stage 1 to the lowest Cu concentration in the intoxication phase. Stage 3 (stabilization stage) lasted from the end of stage 2 to the end of the intoxication phase. The elimination phase made up the fourth toxicokinetic stage (elimination stage) and was characterized by a gradual decrease in Cu concentration.

**Table 1:** Kinetics parameters [accumulation rate constant (k_a_), elimination rate constant (k_e_)] related to Cu total concentrations in *T. castaneum* from the migration experiment (selection environment: non-contaminated medium)

Phase	Stage		NM	IM50	EM20	EM50
I Intoxication	1 Accumulation	k_a1_(day^−1^)	1.439 × 10^−5^A	13.14 × 10^−5^A	1.612 × 10^−5^A	1.562 × 10^−5^A
			(2.122 × 10^−6^–2.667 × 10^−5^)	(0–0.199)	(0–1.126 × 10^−4^)	(0–3.23 × 10^−5^)
		k_e1_(day^−1^)	1.146 × 10^−5^A	1.093 × 10^−5^A	0.467 × 10^−5^A	0.191 A
			(0–0.355)	(5.331 × 10^−6^–1.654 × 10^−5^)	(0–3.872)	(0–0.755)
		R^2^_1_	93.8%	98.6%	70.9%	91%
	2 Depuration	k_a2_(day^−1^)	5.792 × 10^−6^B	15.78 × 10^−6^A	2.239 × 10^−6^B	6.308 × 10^−6^AB
			(5.785 × 10^−6^–5.999 × 10^−6^)	(15.78 × 10^−6^–15.79 × 10^−6^)	(0–6.844 × 10^−6^)	(0–2.397 × 10^−5^)
		k_e2_(day^−1^)	0.297B	0.589A	0.160C	0.214ABC
			(0.297–0.297)	(0.589–0.589)	(0.051–0.269)	(0–0.672)
		R^2^_2_	100%	100%	99.9%	96.6%
	3 Stabilization	k_a3_(day^−1^)	1.343 × 10^−5^A	2.189 × 10^−6^A	8.425 × 10^−4^A	6.042 × 10^−6^A
			(0–5.346)	(0–1.099 × 10^−5^)	(0–6.147 × 10^−4^)	(0–1.946 × 10^−5^)
		k_e3_(day^−1^)	0.309 A	4.094 × 10^−2^A	21.61 A	0.13 A
			(0–1.256)	(0–0.282)	(0–1576 × 10^6^)	(0–0.459)
		R^2^_3_	28.6%	76.7%	31.7%	87.8%
II Elimination	4 Elimination	k_a4_(day^−1^)	8.157 × 10^−4^A	3.102 × 10^−7^A	9.908 × 10^−4^A	8.116 × 10^−4^A
			(0–5.54 × 10^−3^)	(0–9.139 × 10^−7^)	(0–7.103 × 10^−3^)	(0–1.108 × 10^−2^)
		k_e4_(day^−1^)	0.122	0.072	0.113	0.118
			(0–0.457)	(0.02–0.125)	(0–0.556)	(0–0.85)
		R^2^_5_	94.1%	82.4%	84.2%	40.8%

The 95% confidence intervals are shown in brackets

Coefficients of determination (R^2^) describe the proportion of variance in beetle Cu body concentrations explained by the fitted model

Subscripted numbers associated with k and R denote the four stages of the model as follows: *1* the accumulation stage, *2* the depuration stage, *3* the stabilization stage, *4* the elimination stage

*Abbreviations* denote after migration lines (AML): *NM* control lines with no migrants maintained under the same regime as in the main selection experiment, *IM50* lines with 50% ‘internal’ migrants (from the same environment type), *EM20* and *EM50* migrants derived from a line from different selection environment; 20% or 50% of migrants respectively

Differences between the confidence intervals of kinetics parameters (k_a_ and k_e_) are shown as letters (A, B, C) for a single phase, and single constant (A, B, C)

**Table 2:** Kinetics parameters [accumulation rate constant (k_a_), elimination rate constant (k_e_)] related to total Cu concentrations in *T. castaneum* from the migration experiment (selection environment: Cu-contaminated medium)

Phase	Stage		NM	IM50	EM20	EM50
I Intoxication	1 Accumulation	k_a1_(day^−1^)	2.752 × 10^−5^A	1.261 × 10^−5^A	1.846 × 10^−5^A	1.325 × 10^−5^A
			(0–9.75 × 10^−4^)	(0–3.452 × 10^−5^)	(0–6.180 × 10^−5^)	(0–1.281 × 10^−4^)
		k_e1_(day^−1^)	4.814 × 10^−6^A	9.142 × 10^−2^A	1.208 × 10^−5^A	5.547 × 10^−6^A
			(0–1.986 × 10^−5^)	(0–0.673)	(0–15.47)	(0–3.611)
		R^2^_1_	84.1%	89.9%	77%	79.6%
	2 Depuration	k_a2_(day^−1^)	2.024 × 10^−7^A	2.024 × 10^−6^AB	1.874 × 10^−5^B	1.483 × 10^−4^AB
			(0–1.089 × 10^−5^)	(0–2.190 × 10^−5^)	(1.874 × 10^−5^ − 1.874 × 10^−5^)	(0–1.061^−2^)
		k_e2_(day^−1^)	0.055A	0.104A	0.578A	3.321A
			(0–0.306)	(0–0.534)	(0.578–0.578)	(0–237.4)
		R^2^_2_(day^−1^)	99.2%	96.3%	100%	90.6%
	3 Stabilization	k_a3_(day^−1^)	1.468 × 10^−5^A	1.041 × 10^−5^A	9.098 × 10^−6^A	8.577 × 10^−6^A
			(0–6.127 × 10^−5^)	(0–5.927 × 10^−5^)	(0–5.160 × 10^−5^)	(7.728 × 10^−6^–9.385 × 10^−6^)
		k_e3_(day^−1^)	0.263 A	0.214 A	0.206 A	0.181 A
			(0–1.15)	(0–1.288)	(0–1.208)	(0.162–0.199)
		R^2^_3_	55.7%	55%	21.7%	99.9%
II Elimination	4 Elimination	k_a4_(day^−1^)	2.467 × 10^−3^A	5.038 × 10^−8^A	5.298 × 10^−8^A	4.175 × 10^−3^A
			(0–1.527 × 10^−2^)	(0–2.732 × 10 − 7)	(0–1.775 × 10^−7^)	(0–0.015)
		k_e4_(day^−1^)	0.177A	0.043A	0.030A	0.298A
			(0–0.970)	(0–0.077)	(0.005–0.055)	(0–0.960)
		R^2^_5_	79.6%	67.6%	52.9%	94.1%

The 95% confidence intervals are shown in brackets

Coefficients of determination (R^2^) describe the proportion of variance in beetle Cu body concentrations explained by the fitted model

Subscripted numbers associated with k and R denote the four stages of the model as follows: *1* the accumulation stage, *2* the depuration stage, *3* the stabilization stage, *4* the elimination stage

*Abbreviations* denote after migration lines (AML): *NM* control lines with no migrants maintained under the same regime as in the main selection experiment, *IM50* lines with 50% ‘internal’ migrants (from the same environment type), *EM20* and *EM50* migrants derived from a line from different selection environment; 20% or 50% of migrants respectively

Differences between the confidence intervals of kinetics parameters (k_a_ and k_e_) are shown as letters (A, B, C) for a single phase, and single constant (A, B, C)

The rapid increase in body Cu concentration in the first stage of the experiment (Fig. 2) was consistent with reports made by other authors for various metals and invertebrates, such as earthworms (Atkins 1969), insects (Bednarska et al. 2011; Laskowski et al. 2010; Postma et al. 1996), and crustaceans (Soedarini et al. 2012). The physiological basis of this phenomenon in *T. castaneum* may be the immobilization of metals in gut wall in an insoluble form−granules (Hopkin 1989; Postma et al. 1996; Rost-Roszkowska et al. 2010). This process appears to be the first defense mechanism against metal intoxication (Laskowski et al. 2010; Posthuma and Van Straalen 1993; Postma et al. 1996). The second stage showed an abrupt reduction in Cu concentration to the lowest values recorded in the intoxication phase. Similar reductions have been previously found and discussed by other authors (Bednarska et al. 2011; Laskowski et al. 2010; Soedarini et al. 2012). The pattern appears to result from a threshold Cu concentration being reached in the gut epithelium, leading to gut damage (Argasiński et al. 2012) or acclimation, and then to rapid depuration. Herein proposed hypothesis predicts massive metal accumulation in the intestine during the first stage, and an abrupt reduction during the second stage caused by gut cell exfoliation due to the toxic effect of Cu on epithelial cells. This phenomenon has been mechanistically described by a TK cell demography model (TKCD) (Argasiński et al. 2012), and has been described by Postma et al. (1996) for the midge *Chironomus riparius*. In all treatments, our data showed a clear increase in concentration after the second-stage reduction, suggesting that third stage existed in terms of animal physiology (i.e., separate k_a_ and k_e_ values). The slight, yet clear, increase in Cu concentration, and possible stabilization at a level intermediate to the maximum and minimum values, suggests some sort of acclimation. Cu concentration may be controlled efficiently in the contaminated environment by fine-tuning the assimilation and elimination rates. Copper is an essential element and it is generally well regulated by invertebrates. Alternatively, observed fluctuations in body Cu concentration may be a simple side effect of the cycles of gut epithelial cell replacement. This latter explanation is also supported by the mechanistic TKCD model (Argasiński et al. 2012), and by a model proposed by Postma et al. (1996). Laskowski et al. (2010) and Skip et al. (2014) did not distinguish this stage in their three-stage model. The fourth stage in our approach was equivalent to the elimination phase and followed the pattern commonly described in the literature (Bednarska et al. 2011; Janssen et al. 1991; Laskowski et al. 2010). After being placed on the uncontaminated medium, the beetles continuously eliminated excessive Cu.

The one-compartment, two-phase, four-stage approach proposed in this paper fitted our data very well. The three stages of the intoxication phase, described by separate equations within dependently estimated k_a_ and k_e_ values, appear to reflect observed Cu level fluctuations and changes in animals’ physiology that may occur during metal exposure. It is consistent with the description of gut physiology in metal-intoxicated invertebrates provided by Argasiński et al. (2012). The proposed approach can be considered a more detailed version of the three-stage model proposed by Laskowski et al. (2010) and Skip et al. (2014). The demonstrated approach is simplified, with subjectively demarcated breakpoints and four separately fitted equations for the four stages, because of limitations of the conducted experiment. The experiment was originally designed to compare regular two-stage kinetics in different populations of *T. castaneum*. Only after completing the study did it appear that the traditional two-stage model does not fit the data. Hence, the data collected were too scarce to estimate and test more complex models and did not allow for estimating the breakpoints. Nevertheless, the results suggest that the proposed approach can be used also with estimated breakpoints or even extended to a two-compartment model, provided the sampling is more frequent (Skip et al. 2014). To determine whether the observed changes in metal concentration can be indeed explained by cycles of gut epithelial cell shedding and replacement, detailed studies on the invertebrates’ detoxification physiology are needed.

During the intoxication phase food avoidance can be the first defense mechanism against food contamination. The reduction of consumption in intoxication phase could result in body mass loss and indirectly influence copper body burden. Although direct measure of consumption rate was not available for the present experiment. We compared dry body mass of the beetles sampled at the beginning of each TK stage of the intoxication phase and at the end of intoxication phase in day 28 (two-way ANOVA for treatment and time with outlier detection; data distribution tested for normality and variance homogeneity). The results did not indicate any effect of time on body mass during the intoxication phase (*p* = 0.053; F_3,288_ = 2.656) but significant treatment effect was found (*p* < 0.001; F_7,288_ = 4.33). Interaction was not significant (*p* = 0.965; F_21,288_ = 0.512). It can be, thus, assumed that if dry body mass of the beetles did not change during the experiment, they were not starving and consumed both the contaminated and uncontaminated food similarly. The significant treatment effect indirectly pointed on inbreeding depression: individuals derived from lines that obtained migrants (internal and external) were heavier.

Few studies have investigated differences in metal toxicokinetics between metal adapted and non-adapted populations. In a population of Cd-adapted midges (*Chironomus riparius*), the net accumulation of Cd was lower, the excretion efficiency and equilibrium level were higher (Postma et al. 1996). In the springtail *Orchestella cincta*, adaptation to Cd was manifested as a higher excretion rate caused by an alteration in gut physiology (Posthuma and Van Straalen 1993). Accordingly, we expected immigrants originating from a different line type to influence k_a_ and k_e_ values. Comparison of the confidence intervals for the TK parameters (Tables 1, 2), along with an analysis of the graphs (Fig. 2), indicated that differences between treatments in k_a_ and k_e_ values were negligible. We found significant differences in k_a_ and k_e_ values in the second stage (depuration) in lines from non-contaminated selection environment (Table 1). Accumulation rates for IM50, AML and EM20, AML differed significantly from accumulation rate for NM, AML. Elimination rates were different for three AML: NM, IM50 and EM20 (Table 1). For lines originating from Cu-contaminated selection environment significant difference was found only for k_a_ in the second (depuration) stage: k_a_ value was higher in EM20 lines than in NM lines (Table 2). It is reasonable to assume that the differences were due to changes in gut physiology (Posthuma and Van Straalen 1993; Posthuma et al. 1996): storage capability and excretion efficiency (Cu intake, accumulation in granules and gut epithelium exfoliation). The highest k_a_ value reached by IM50 lines indicates that a high rate of migration could be beneficial to Cu uptake rate and bonding. Indirectly, it suggests that inbreeding depression for Cu management in NM lines is manifested by lowered Cu influx and storage abilities. It is difficult to compare this value to the value estimated for the EM50 treatment because of lower goodness-of-fit (R^2^ = 96.6%), and wider (0.0–23.97 × 10^−5^) CI (confidence interval) overlapping the CI for the NM treatment (Table 1). Also the IM50 lines had the highest elimination rate significantly higher than for NM lines and for EM20 lines. Because the CI was widest for EM50 lines (0.000–0.672 day^−1^; R^2^ = 96.6%) and overlapped with the CIs of the other treatments it is impossible to discuss effects of external immigrants. This result again indirectly suggests that inbreeding depression for Cu management in NM lines is manifested as less efficient Cu excretion.

The duration of subsequent stages during the intoxication phase should be also analyzed and discussed in terms of gut physiology and defense mechanisms combined with gut epithelium exfoliation. Nonetheless, it is difficult to find a clear pattern for differences between treatments (groups). The difference that was most pronounced was the prolongation of the second (depuration) stage in lines from non-contaminated environment reinforced with migrants coming from copper-contaminated environment from 4 days in NM AML to 6 days in EM20 AML and 8 days in EM50 AML (Fig. 2). The duration of this stage in NM AML lines from copper-contaminated environment was 6 days. It can be stated that depuration process after reaching the maximal copper body burden during the intoxication phase was longer in lines derived from contaminated environment and this trait was transferred to lines from non-contaminated environment by migrants. The physiological mechanism of the observed phenomenon can be higher resistance to copper intoxication (via gut epithelium) of the beetles derived from the contaminated environment followed by slower gut exfoliation and removal of the deposited copper.

The lack of unequivocal influence of migrants in the lines from Cu-contaminated selection environment indirectly suggests that the lines suffered less from inbreeding and contamination than lines derived from non-contaminated environment, or that inbreeding depression for Cu management was so strong that migrants were not able to enhance the investigated traits (Tables 1, 2). The former hypothesis would suggest a beneficial influence of mild Cu stress on inbred populations. Marr et al. (2006) showed that in a population of wild song sparrows, inbreeding depression had a lesser influence on laying date in colder years. Dahlgaard and Hoffmann (2000) showed that heat stress had no effect on inbreeding in fruit flies. Therefore, we cannot reject the hypothesis that intermediate levels of Cu-contamination could mitigate inbreeding depression in *T. castaneum*. This hypothesis is in stark contrast to the stress hypothesis which assumes that inbreeding depression is enhanced by harsh environments. Armbruster and Reed (2005) observed this trend empirically in 75% of the cases included in their metaanalysis but they also observed counter examples. However, in certain circumstances this may not be the case.

Furthermore, it must be noted that significant differences were found for the TK stage with the highest goodness-of-fit and the narrowest CI (R^2^ values 96.6%–100%; Table 1). This finding indicates that TK experiments must be very densely sampled and the models must show high goodness-of-fits if TK models are to be a useful tool for comparing different treatments.

## Electronic Supplementary Material

Below is the link to the electronic supplementary material.

Supplementary material 1 (DOCX 12 KB)
