# Supplementary material for: A new TK model approach to assess the effect of migration on copper toxicokinetics in inbred populations of the flour beetle, Tribolium castaneum
Source: Bull Environ Contam Toxicol. 2017 May 26;99(1):9–16. doi: 10.1007/s00128-017-2093-7 (PMC5487540; doi:10.1007/s00128-017-2093-7)
Supplement: Supplementary file 1 — Supplementary material 1 (DOCX 12 KB) [file 128_2017_2093_MOESM1_ESM.docx]

**A new TK model approach to assess the effect of migration on copper toxicokinetics in inbred populations of the flour beetle, *Tribolium castaneum***

Sebastian Żmudzki*; Natnael T. Hamda^§^; Patrycja Gibas*

Supplemental data

After 22 generations, 20 lines were selected for the study from among the 200 inbred lines: ten of them from lines kept in non-contaminated selection environment and the other 10 from lines kept in Cu-contaminated selection environment. Prior to selection, all of the lines were screened for the effects of Cu contamination on reproduction rate. We randomly selected 20 fertile pupae of each sex from each line. The sexes were separately maintained in the appropriate experimental conditions until maturation. The beetles used in the experiment were 59 - 62 days old (from oviposition). For each of 200 lines, 3 groups consisting of 10 individuals each (five females and five males) were established. All of the groups were then placed into 20 ml containers prepared with 2 g of the same medium used in selection experiment (0 mg kg^-1^ Cu or 1000 mg kg^-1^ Cu) for 3 days. The beetles copulated and the females laid eggs. After 3 days the animals were removed and 14 g of medium was added to each container and mixed by shaking for 15 s. The added medium was adequately contaminated (0, 1000, 1143, 3286 and 3429 mg kg^-1^) to obtain the following Cu contamination levels (one replicate for every line): 0, 1000, and 3000 mg kg^-1^ Cu. The progeny that emerged in every container were counted for several subsequent days, until the last live individual had emerged. Reproduction rate F ratio as measure of Cu resistance was used. It was calculated as: F_Cu1000_ = N_Cu1000_ N_Cu0_^-1^ and F_Cu3000_=N_Cu3000_ N_Cu0_^-1^, where N is the number of mature, surviving offspring produced per female per day, and the indices denote the medium contamination level (i.e., Cu0 – non-contaminated medium, Cu1000 – medium contaminated with 1000 mg kg^-1^ Cu, and Cu3000 – medium contaminated with 3000 mg kg^-1^ Cu). The 10 populations with the lowest F_Cu1000_ and F_Cu3000_ values were chosen from among the 100 inbred lines maintained in non-contaminated selection environment. Conversely, the 10 populations with highest F_Cu1000_ and F_Cu3000_ values were selected from among the 100 strains maintained in the Cu-contaminated selection environment. Thus, the lines that were selected for the main experiment were the 10 least Cu-resistant and 10 most Cu-resistant of the 200 inbred lines. We compared the F_Cu1000_ and F_Cu3000_ values for the selected lines (Cu-adapted and non-adapted) with a one-way ANOVA. The ANOVA revealed significant differences between the two groups (p<0.05). Lines from the two groups were pair-matched (Cu-exposed line and non-Cu-exposed control line) for migration experiment. The matching criterion was to achieve the highest possible differences between control and Cu-exposed populations in F_Cu1000_ and F_Cu3000_ for all pairs, expressing the difference between lines in terms of Cu resistance.
